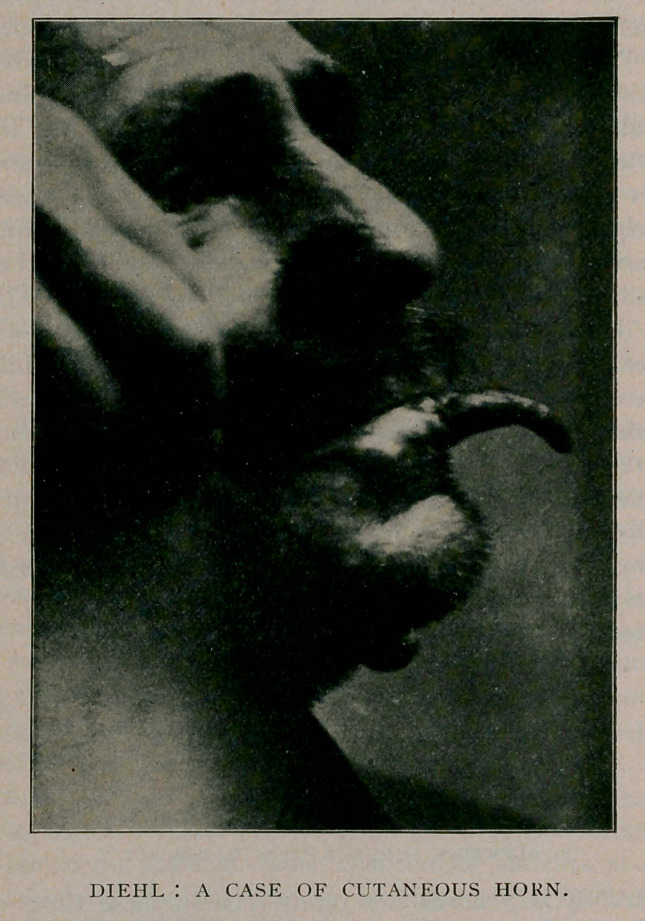# A Case of Cutaneous Horn

**Published:** 1902-12

**Authors:** Alfred E. Diehl

**Affiliations:** Buffalo, N. Y., 362 Pearl Street.


### CLINICAL REPORT.

### A Case of Cutaneous Horn.

By ALFRED E. DIEHL, A. M., M. D., Buffalo, N. Y.

CUTANEOUS horns are rarely seen in these times on the human subject and are regarded merely as curiosities. They present an outward resemblance to the horns of animals, but arise from the skin instead of from the bone, have not their extreme hardness and are not of a uniform shape or size. Horns are usually solitary, but may be multiple; they are either laminated or fibrillated, solid, hard and dry, rounded, oval or angular, generally bent or twisted, and of a gray, yellow, brown, green or black color. They are largest at the base and vary in size from a quarter of an inch to twelve inches in length; occasionally they are branched. The great majority occur on the head and face; half the cases occur on the scalp, forehead and temples; next in frequency they occur on the eyelids, ears and nose, glans and testicle, the remainder on the body, their preference for the seborrheic regions of the skin being somewhat

DIEHL : A CASE OF CUTANEOUS HORN.

remarkable. They occasion no subjective symptoms and are painful only when injured. The treatment is very simple, consisting merely in removal, followed by thorough curettement and cauterisation of the base, otherwise there will be a recurrence. Lebert who collected the most comprehensive data on this subject observed that 12 per cent., of the cases develop into epitheliomata, hence the necessity for their thorough removal.

The situation of the horn in this case is unusual. I have been unable to find a reference to a similar one, that is, where it has grown from the lip. The history of this case is as follows:

E. H., male, aged 45 years, came to me five years ago presenting an epithelioma on the lip, which I excised, the scar of which can be seen in the photograph on the side of the lip opposite to that occupied by the horn. Three years later he again came to me with a small horn on the lip, but which he would not allow me to remove; finally, it grew to such dimensions that it became a nuisance to him, and he allowed me to remove it, which was done under local cocain anesthesia, followed by curetting and cauterisation. The appearance of the horn is as follows: length, 2 inches; at about the middle, it shows a distinct branch; the base is oval, $\frac{3}{4}$ of an inch in its greatest diameter; it is of a brown color, fibrillated and curved upon its under surface; the interior of the base was of a soft, friable papillomatous nature, which indicated that the origin of the horn was most probably an ordinary wart.

362 Pearl Street.

## Figures and Tables

**Figure f1:**